# Effect of multiple micronutrient supplementation on survival of HIV-infected children in Uganda: a randomized, controlled trial

**DOI:** 10.1186/1758-2652-13-18

**Published:** 2010-06-03

**Authors:** Grace Ndeezi, Thorkild Tylleskär, Christopher M Ndugwa, James K Tumwine

**Affiliations:** 1 Department of Paediatrics and Child Health, School of Medicine, College of Health Sciences, Makerere University, Kampala, Uganda; 2 Centre for International Health, University of Bergen, Norway

## Abstract

**Background:**

Micronutrient deficiencies compromise the survival of HIV-infected children in low-income countries. We assessed the effect of multiple micronutrient supplementation on the mortality of HIV-infected children in Uganda.

**Methods:**

In a randomized, controlled trial, 847 children aged one to five years and attending HIV clinics in Uganda were stratified by antiretroviral therapy (ART, n = 85 versus no ART, n = 762). The children were randomized to six months of either: twice the recommended dietary allowance of 14 micronutrients as the intervention arm (vitamins A, B_1_, B_2_, niacin, B_6_, B_12_, C, D and E, folate, zinc, copper, iodine and selenium); or the standard recommended dietary allowance of six multivitamins (vitamins A, D_2, _B_1_, B_2_, C and niacin) as a comparative "standard-of-care" arm. Mortality was analyzed at 12 months of follow up using Kaplan Meier curves and the log rank test.

**Results:**

Mortality at 12 months was 25 out of 426 (5.9%) children in the intervention arm and 28 out of 421 (6.7%) in the comparative arms: risk ratio 0.9 (95% CI 0.5 - 1.5). Two out of 85 (2.4%) children in the ART stratum died compared with 51 out of 762 (6.7%) in the non-ART stratum. Of those who died in the non-ART stratum, 25 of 383 (6.5%) were in the intervention arm and 26 of 379 (6.9%) in the comparative arm; risk ratio 1.0 (95% CI 0.6 - 1.6). There was no significant difference in survival at 12 months (p = 0.64, log rank test). In addition, there was no significant difference in mean weight-for-height at 12 months; 0.70 ± 1.43 (95% CI 0.52 - 0.88) for the intervention versus 0.59 ± 1.15 (95% CI 0.45 - 0.75) in the comparative arm. The mean CD4 cell count; 1024 ± 592 (95% CI 942 - 1107) versus 1060 ± 553 (95% CI 985 - 1136) was also similar between the two groups.

**Conclusions:**

Twice the recommended dietary allowance of 14 micronutrients compared with a standard recommended dietary allowance of six multivitamins for six months was well tolerated, but it did not significantly alter mortality, growth or CD4 counts. Future intervention studies should carefully consider: (1) the composition and dosing of the supplements; and (2) the power needed to detect a difference between arms.

**Trial Registration:**

ClinicalTrials.gov Identifier: NCT00122941

## Background

Mortality in HIV-infected children living in low-income countries is still high compared with high-income countries [1,2]. Malnutrition in children under five years of age is highly prevalent and both macro and micronutrient deficiencies are likely to co-exist [3-6], especially among HIV-infected children.

Micronutrients are important for maintaining optimal functioning of the individual's immune response. Selenium and vitamin E are involved in the maintenance of the oxidant defence system, while zinc and vitamin A play a significant role in maintaining cellular integrity [7]. Vitamin B_12 _is important in the formation of proteins and proper functioning of a large number of enzymes and the immune system [8]. Deficiency of the important components of the endogenous anti-oxidant defence system leads to accumulation of oxidative stress, including oxidative damage [9]. Vitamin A and zinc deficiencies are associated with increased susceptibility to infections, increased severity of illness and mortality [10,11].

Studies in HIV-infected children have demonstrated that multiple, large doses of vitamin A reduces diarrhoea episodes, increases CD4 count and reduces all-cause mortality [12-14]. Zinc supplementation in children whose HIV status was not known in Asia and Latin America reduced the incidence, duration and severity of diarrhoea and pneumonia episodes [15]. In a study of efficacy and safety of zinc, mortality was lower in the zinc-supplemented group [16]. Most of these studies were single micronutrient interventions, yet deficiencies are less likely to exist singly: hence the efforts to provide multiple micronutrients as opposed to single nutrient supplements in both children and adults studies [17-21].

To achieve normal plasma levels of micronutrients, HIV-infected adults required multiples of the recommended dietary allowance (RDA) compared with HIV-negative men. Those consuming adequate recommended intake had a relatively high prevalence of deficiencies compared with uninfected adults with similar intake [22]. Multivitamin supplementation using multiples of the RDA resulted in reduction of progression to Stage 4 disease and mortality in pregnant and lactating women in Tanzania [13].

Supplementation with multiple micronutrients had no effect on mortality in one study of adults with HIV [23], while it reduced mortality in another trial that used multiples of RDAs in patients with CD4 counts of less than 200/mm^3 ^[18]. Hitherto, there are no published studies that have examined the effect of multiple micronutrient supplementation on mortality of HIV-infected children.

The objective of this study was to assess the efficacy of a supplement containing twice the RDA (2 RDA) of 14 multiple micronutrients on mortality of HIV-infected children in Uganda, and to document any adverse effects associated with this dosing of multiple micronutrients. We hypothesized that daily administration of twice the recommended dietary allowance of multiple micronutrients to HIV-infected children aged one to five years for six months would reduce all-cause mortality from 24% to 14.4% in one year.

## Methods

### Study design, site and population

This was a randomized, controlled, double-blind, hospital-based trial of a supplement containing twice the RDA (2 RDA) of 14 micronutrients (minerals and vitamins) versus a formula of six multivitamins at the standard RDA (1 RDA) dose administered to HIV-infected children for a period of six months and followed up to 12 months. The trial was conducted between June 2005 and June 2008 in Uganda at: the Paediatric HIV clinics of the national referral hospital (Mulago); two private, not-for-profit centres (Nsambya Hospital and Mildmay Centre) in Kampala, the capital city; and four regional hospitals (Mbale, Mbarara, Masaka, Lira).

At each site, a paediatrician was in charge of the study and worked with a nurse and laboratory technician, who had undergone training on study procedures. The principal investigator initiated the study at all the sites and supervised data collection once a week at the Kampala (central) sites and once every four weeks at the regional hospitals (rural sites). Similar operating procedures were followed.

The principal investigator or another paediatrician enrolled children aged one to five years whose mothers or caretakers gave informed written consent to participate and who had attended the clinic at least once. The mothers or caretakers also had to adhere to a regular study follow-up schedule for one year. Their HIV status had earlier been confirmed by either an antibody test or DNA-PCR if younger than 18 months of age. These children were stratified into two groups: those receiving antiretroviral drugs (ARVs) and those not yet started on antiretroviral therapy (ART). Children enrolled in other studies, those residing more than 15 kilometres from the clinic and those whose parents or caretakers were anticipating moving from the study area were excluded.

The study was approved by Makerere University College of Health Sciences Research and Ethics Committee, the participating hospitals, the Uganda National Council for Science and Technology and the Regional Committee for Medical Research Ethics, Western Norway. Counselling for initiation of ART and adherence was offered to all the participants, while ongoing adherence counselling was provided to those who were already receiving ART. Initiation of ART, treatment for concurrent illnesses and prophylaxis was offered according to the World Health Organization (WHO) and national paediatric HIV management guidelines.

### Micronutrient supplements

The trial supplements were manufactured in the form of a white powder, packaged in plastic containers and serially labelled according to strata (S1 or S2). The intervention supplement consisted of twice the recommended dietary allowance (2 RDA) of vitamins A, B_1_, B_2_, niacin, B_6_, B_12_, C, D and E, folate, zinc, copper, iodine and selenium; the comparative "standard-of-care" supplement consisted of the RDA (1 RDA) of vitamins A, C, D, B_1_, B_2 _and niacin (Table 1).

**Table 1:** The formulation of intervention supplement and the comparative "standard-of-care" supplement used in the supplementation trial of HIV-infected children, Uganda

Micronutrient	Intervention arm 2 RDA	Comparative arm "standard-of-care" 1 RDA
Vitamin A (mcg)	800	400
VitaminB1 (mg)	1.2	0.6
Vitamin B2 (mg)	1.2	0.6
Niacin (mg)	1.6	0.8
Vitamin B6 (mg)	1.2	-
Vitamin B12 (mcg)	2.4	-
Vitamin C (mg)	50	25
Vitamin D (IU)	400	200
Vitamin E (mg)	14	-
Folate (mcg)	400	-
Selenium (mcg)	60	-
Zinc (mg)	10	-
Copper (mcg)	800	-
Iodine (mcg)	180	-

The comparative "standard-of-care" supplement was designed to be similar to the regular multivitamin tablet supplied as the standard of care at paediatric HIV clinics in Uganda. Upon administration to the child, both supplements were dissolved in milk or water. The nurse demonstrated how to prepare a dose and allowed the mother to prepare and administer the first dose in the clinic. Each participant received 140g (one container) per month, which was equivalent to 35 doses.

### Randomization and blinding

The eligible participants were randomized to either the intervention or "standard-of-care" in two strata. A WHO officer in Geneva, who was not part of the study team, generated the randomization code in permuted blocks of 4 to 20 using the Stata software. The list was sent to NUTRISET (France), which manufactured the trial supplements and packaged them in serially labeled identical containers. The consistency of the powder, colour and smell were similar. All investigators, staff and parents or caretakers were blinded to treatment assignment. The randomization code was made available to the investigators upon completion of data collection.

### Clinical and laboratory assessment

Mothers or caretakers were interviewed about the children's previous medical, nutritional history and presenting symptoms. The participants underwent a detailed physical examination, including anthropometry and WHO staging for paediatric HIV/AIDS. Weight was taken using a scale (uniscale 01-410-15) to the nearest 0.1kg, and height was taken to the nearest 0.1 centimetre using a portable infant-child length-height measuring board (Shorr productions, Olney, Maryland, USA). A plastic tape was used to measure mid-arm circumference to the neatest 0.1cm. HIV/AIDS clinical disease staging was decided using clinical signs against the WHO classification for paediatric HIV [24].

Two millilitres (ml) of blood was collected in a 5ml EDTA vacutainer tube (Becton Dickinson, Franklin Lakes, N.J.) by venipuncture from the cubital fossa or dorsum of the hand, and was analyzed for a complete blood count (Act 5Diff instrument Beckman Coulter ) and CD4 cell count (FACScan instrument and MultiSET software Beckton Dickinson). C-reactive protein (CRP) was analyzed using a qualitative method (Human Gesellschaft fur Biochemica und Diagnostica mbH, Germany). Agglutination indicated a C-reactive protein of more than 6mg/L and this was reported as a positive CRP. An additional 3-5ml blood sample was collected and serum was analyzed for zinc and other trace elements using inductively coupled atomic emission spectrometry (ICP-AES) [25].

Participants were followed at the clinics monthly for the first six months, and at nine and 12 months. A record of illness, anthropometry, physical examination findings, investigations and treatment was kept for each visit. Parents or caretakers were requested to report to the clinic whenever the child got sick, was admitted to hospital or died. Those admitted were followed until discharge, and if they died, information was recorded on a mortality and adverse event form. Information on missed doses of the supplement was recorded on each monthly visit for six months.

Compliance was assessed by measuring the remaining supplement using a light-weight weighing scale (Philips HR 2389/B 9.OV/DC). Each study participant was expected to take 4g of the supplement per day (one scoop) and this was equivalent to 120g in 30 days. A proportion of the amount of supplement taken against the expected was used as a measure of compliance. Overall compliance was assessed at the end of six months, whereby the average compliance was derived by adding the compliance rates on all the six scheduled visits.

After six months, no study supplements were given, but the children received the regular multivitamin supplements from the clinic as the standard of care. Follow up to 12 months was to ascertain whether there was any sustained effect of the intervention. Children who were not brought for scheduled visits were traced by telephone or physically by the health visitor. Those who missed more than two scheduled visits and could not be traced were declared lost.

### Outcomes

Information on mortality was collected from verbal reports by the parents or caretakers or from hospital records. Those who died at home were reported by telephone or through tracing. Side effects attributed to the supplement were assessed and recorded at the monthly visits. A serious adverse event (SAE) form was completed if an adverse event had occurred. Conditions that resulted in hospitalization, required medical intervention to prevent a serious outcome, or were life threatening or fatal were regarded and reported as SAEs. The paediatrician decided on the relationship to the intervention using a set of conditions or known side effects of micronutrients. All SAEs warranted stoppage of the trial supplement when closely related to the intervention.

### Statistical issues

The estimated sample size of 411 children in each arm was based on data from two studies. The first assumption was a mortality rate in the comparative arm of 24% in one year, based on the mortality rate in a study conducted in Mulago Hospital, Kampala, before highly active antiretroviral therapy (HAART) was available to HIV-infected children [26]. The mortality among HIV-infected children occurring between the ages of 12 and 25 months was close to 24%. We assumed a similar mortality in the whole age span of one to five years.

The second assumption - that all-cause mortality would be reduced to 14.4% (a 40% reduction) - was based on a study in Tanzanian children aged six months to five years where supplementation with vitamin A was associated with a 49% reduction in overall mortality and 63% among HIV-infected children [13]. We decided to use the 40% reduction level as we anticipated improved general care of HIV-infected children over time. Finally, we used a precision of 5%, and 95% confidence interval. The power estimate was 90% with an assumption of 10% loss to follow up.

Weight-for-height (WHZ), height-for-age (HAZ) and weight-for-age (WAZ) z-scores were computed using the WHO International Growth References [27]. Sub-group analysis was based on whether the children were receiving ART or not.

Statistical analysis was performed using SPSS version 15.0. Baseline characteristics were compared in the two treatment groups using proportions, and differences were tested with the Chi-square or Fisher's Exact test. To determine the association between patients' characteristics and mortality, logistic regression was used. Risk ratios and 95% confidence intervals were used to test the strength of association. Comparisons of treatment efficacy were analyzed on intention-to-treat basis in the two arms. Kaplan Meier curves and the log rank test were used to compare survival in the two arms.

## Results

**Figure 1:**
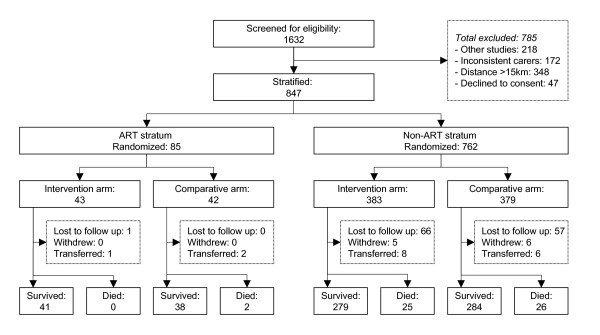
**Trial profile**.

A total of 1632 children aged 12 to 59 months attending paediatric HIV clinics at the study sites were screened for eligibility (Figure 1). Out of the 847 children enrolled, 704 (83.1%) were from the study sites in Kampala (the capital city); the rest were from the rural sites.

### Baseline characteristics of participants

Almost equal proportions of children were assigned to the two arms in both strata. More than half of the children (470/847, 56%) were aged less than 36 months. Table 2 shows the baseline characteristics and these were comparable in the two groups.

**Table 2:** Baseline characteristics of the 847 Ugandan HIV-infected children aged 1-5 years enrolled in the micronutrient supplementation trial by ART stratum

Characteristics	ART stratum (n = 85)			Non-ART stratum (n = 762)		
	Intervention arm n = 43 (%)	Comparative arm n = 42 (%)	p-value	Intervention arm n = 383 (%)	Comparative arm n = 379 (%)	p-value
Age <36 months	8 (8.6)	16 (38.1)	0.06	219 (57.2)	227 (59.9)	0.46
Male	20 (46.5)	23 (54.8)	0.52	201 (52.5)	182 (48.0)	0.25
Caretaker: mother	24 (55.8)	21 (50.0)	0.67	278 (72.6)	278 (73.4)	0.87
Study site: Kampala	40 (93.0)	40 (95.2)	1.00	313 (81.7)	311 (82.1)	0.93
Fever	9 (20.9)	8 (19.0)	1.00	76 (19.8)	81 (21.4)	0.65
Diarrhoea	1 (2.3)	2 (4.8)	0.62	42 (11.0)	51 (13.5)	0.32
Cough	27 (62.8)	26 (61.9)	1.00	220 (57.4)	219 (57.8)	0.94
Persistent diarrhoea	0 (0.0)	0 (0.0)	-	11 (26.8)	11 (23.4)	0.81
Cotrimoxazole prophylaxis	43 (100.0)	42 (100.0)	-	340 (88.8)	334 (88.1)	0.82
Routine multivitamins	18 (41.9)	13 (31.0)	0.37	241 (62.9)	219 (57.8)	0.16
Vitamin A in past 6 months	20 (46.5)	16 (38.1)	0.51	183 (47.8)	175 (46.2)	0.66
WHO Stage 3 or 4	-	-	-	124 (33.4)	114 (30.1)	0.53
WHZ less than -2 z score	2 (4.6)	3 (7.1)	1.00	53 (13.8)	50 (13.2)	0.75
HAZ less than -2 z score	22 (51.2)	23 (54.7)	1.00	190 (49.6)	197 (51.9)	0.65
WAZ less than -2 z score	5 (11.6)	6 (14.3)	1.00	110 (8.7)	125 (32.9)	0.24
Current hospitalization	1 (0.02)	3 (7.1)	0.36	27 (7.0)	16 (4.2)	0.12
Ever hospitalized	25 (58.1)	27 (64.3)	0.66	226 (59.0)	200 (52.8)	0.09
CRP positive (n = 565)	15 (24.6)	11 (18.0)	0.65	122 (24.1)	119 (23.5)	0.70
CD4% <20 (n = 720)	10 (27.0)	8 (24.2)	1.00	124 (38.4)	136 (41.6)	0.42
Zinc <10 μmol/L (n = 336)	5 (26.3)	14 (73.7)	0.01	86 (50.6)	84 (49.4)	0.62

### Follow up

All the study participants took 85% or more of the study supplements. Adverse effects were reported in 16 children (1.9%) and these included vomiting in 12 children and diarrhoea in four children. Of the 12 children who vomited, 6/426 (1.4%) were in the intervention arm and 6/421(1.4%) in the comparative arm. These symptoms were minor and did not warrant stopping the supplement. There were no other adverse effects attributed to the intervention.

By 12 months, 124 (14.6%) children were lost to follow up: 67/426 (15.7%) in the intervention arm and 57/421(13.5%) in the comparative arm. Most of the participants lost to follow up (90/124; 72.5%) were from the central region (Kampala sites). However, the proportion of loss to follow up was higher in the regional than the central sites (23.8% vs. 12.8%; p < 0.01). Children who were not receiving cotrimoxazole routinely (22.7% vs. 13.7%; p = 0.04), CRP positive (13.5% vs. 9.0%; p = 0.00) and those who were underweight (19.9% vs. 12.1%; p = 0.01) were also more likely to be lost to follow up. The other characteristics were similar to those who completed the study.

### Mortality

The overall mortality at 12 months of follow up was 53/847 (6.3%). In the intervention arm, the mortality was 25/426 (5.9%) and in the comparative arm 28/421 (6.7%). The difference between the arms was not statistically significant; risk ratio 0.9 (95% CI 0.5 - 1.5). In the ART stratum, two out of the 85 (2.4%) children died compared with 51/762 (6.7%) in the non-ART stratum. Of those who died in the non-ART stratum, 25/383 (6.5%) were in the intervention arm and 26/379 (6.9%) in the comparative arm; risk ratio 1.0 (95% CI 0.6 - 1.6).

**Figure 2:**
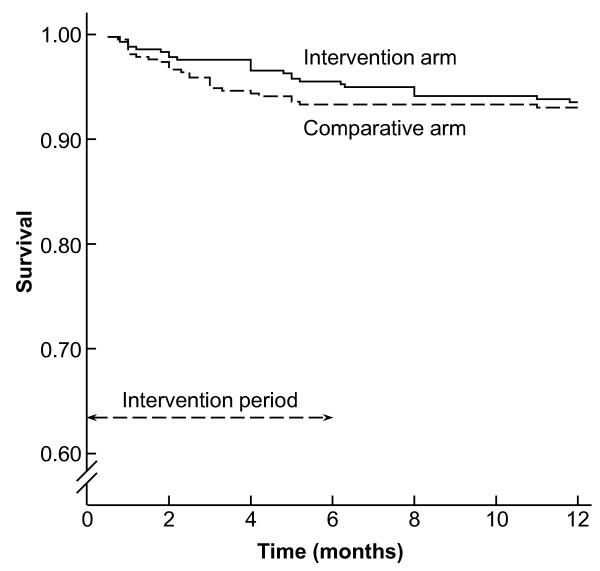
**Kaplan-Meier survival curves of the two arms of the supplementation trial in HIV-infected children at 12 months of follow up**.

Figure 2 shows the Kaplan Meier probability of survival by arm. There was no significant difference in survival at 12 months of follow up (log rank statistic 0.22, 1df, p-value 0.64). The mean survival time was 10.6 months (95% CI 10.3 - 10.9) in the intervention arm and 10.7 (95% CI 10.4 - 11.0) in the comparative arm. The mean survival time was 11.6 months in the ART (95% CI 11.3 - 12.0) stratum and 10.5 (95% CI 10.3 - 10.8) in the non-ART stratum.

On bivariate analysis for baseline characteristics, presence of fever, diarrhoea, cough, hospitalization at enrolment, low anthropometric indices below minus 2 z-scores, and WHO Stage 3 or 4 disease were associated with a shorter time of survival (Table 3). Those who were taking cotrimoxazole routinely within one month before the study had better survival. Other baseline characteristics and routine multivitamin supplementation were not significantly associated with mortality. At multivariate analysis, presence of fever, hospitalization at enrolment visit, WHO Stage 3 or 4, and being underweight independently predicted early mortality.

**Table 3:** Factors associated with mortality in HIV-infected children aged 1-5 years enrolled in the micronutrient supplementation trial in Uganda

Baseline characteristic	Unadjusted hazard ratio (95%CI)	p-value	Adjusted hazard ratio (95%CI)	p-value
Fever	3.7 (2.1 - 6.3)	<0.001	2.1 (1.1 - 3.9)	0.02
Diarrhoea	2.0 (1.0 - 3.9)	0.05	1.4 (0.6 - 2.9)	0.42
Cough	2.1 (1.1 - 3.8)	0.02	1.8 (0.9 - 3.5)	0.08
Hospitalization (current)	6.9 (3.6 - 13)	<0.001	2.6 (1.2 - 5.7)	0.02
Routine cotrimoxazole	0.4 (0.2 - 0.8)	0.01	0.4 (0.2 - 0.9)	0.03
Vitamin A in past 6 months	1.4 (0.8 - 2.4)	0.02	1.3 (0.7 - 2.3)	0.39
WHZ score <-2	3.3 (1.8 - 6.0)	<0.001	0.8 (0.4 - 1.7)	0.62
WAZ score <-2	5.4 (3.0 - 9.8)	<0.001	2.6 (1.2 - 5.8)	0.02
WHO Stage 3 or 4	5.1 (2.9 - 9.0)	<0.001	2.9 (1.5 - 5.6)	<0.01

The most common cause of death was pneumonia accounting for 20/53 (37.7% )of the deaths: eight of the 20 children died of severe pneumonia, six of severe acute malnutrition with severe pneumonia, three of pnemocystis jiroveci pneumonia and a further three died of measles with severe pneumonia. Acute febrile illness and malaria accounted for 11/53 (20.8%) of the deaths, including eigth deaths due to acute febrile illness/malaria, two to cerebral malaria and one child died of malaria with severe anaemia.

Other causes of death were acute diarrhoea with dehydration (6/53; 11.3%), persistent diarrhoea with dehydration (3/53; 5.7%), measles with other complications (3/53; 5.7%), severe acute malnutrition with tuberculosis (2/53; 3.8%), pyogenic meningitis (2/53; 3.8%), cryptococcal meningitis (1/53; 1.9%), and Kaposi's sarcoma (1/53; 1.9%). In 4/53 (7.5%) children, the cause of death could not be established. Seven children died outside the hospital, including the four whose cause of death could not be ascertained.

### Effect of multiple micronutrient supplementation on anthropometry and CD4 cell count

Multiple micronutrient supplementation had no effect on anthropometry as shown in table 4. CD4 cell count was available for 399 surviving children at one year of follow up. Of 195 children who had received twice the RDA of multiple micronutrients, 55 (28.2%) had CD4 cell counts <20% compared with 46/204 (22.5%) who received the standard of care. This difference was not significant (OR 0.74; 95% CI 0.74 - 1.17), implying that the intervention did not have an impact on CD4 count.

**Table 4:** The effect of multiple micronutrient supplementation on anthropometry and CD4 cell count in HIV-infected children aged 1-5 years

Measurement at 12 months	Intervention arm 2 RDA of 14 micronutrients		Comparative arm 1 RDA of 6 multivitamins "standard-of-care"		
	**Mean (SD)**	**95% CI**	**Mean (SD)**	**95% CI**	**p value**
Weight-for-height (WHZ)	0.70 (1.43)	0.52 to 0.88	0.59 (1.15)	0.45 to 0.75	0.39
Height-for-age (HAZ)	-2.17 (1.60)	-2.37 to -1.95	-2.42 (1.50)	-2.61 to -2.23	0.08
Weight-for-age (WAZ)	-0.78 (1.30)	-0.96 to -0.62	-0.97 (1.03)	-1.11 to -0.84	0.07
CD4 count	1024 ( 592)	942 to 1107	1060 ( 553)	985 to 1136	0.53

## Discussion

Twice the recommended dietary allowance of 14 micronutrients given to HIV-infected children daily for six months showed no significant difference in all-cause mortality at 12 months of follow up compared with the "standard-of-care" of the RDA of six multivitamins. This lack of effect may be real. However, it might also be due to other factors.

The first possible reason for "no effect" is that we did not provide a true placebo since multivitamin supplementation was the standard of care in the paediatric HIV clinics in Uganda.

The second reason is the supplement composition and the dosage. In this study, we included 14 of the micronutrients judged to be vital and these were provided in the 2 RDA arm. This was done to minimize the risk of toxicity. But perhaps the individual vitamins and minerals should have been dosed higher, at least for those whose therapeutic window was wide [28]. In our supplement, we did not include iron, based on earlier studies that suggested that iron was potentially detrimental in HIV patients [29].

The third issue is the duration of supplementation and follow up, which have been variable in several studies. Although not significant, there is a divergence of the survival curves during the first six months with supplements and a convergence during follow up without supplementation. Would a longer supplementation time in a larger cohort have demonstrated an effect?

The fourth issue is that the study was designed at a time when the mortality due to HIV was high and few children had an opportunity to receive ART [30]. The mortality figures in these clinics improved continuously over time due to improved care and access to antiretroviral therapy. This led to a lower mortality in the study than anticipated at the design stage.

As previously reported in sub-Saharan Africa, the mortality was lower in children who were already receiving ART at the time of enrolment compared with those who were not. This was comparable to mortality reported in children receiving HAART by other researchers in sub-Saharan Africa [31]. The factors associated with mortality in the regression model included low weight for age, advanced HIV disease (WHO Stage 3 and 4) and hospitalization at enrolment. These findings are not surprising since other studies have reported that malnutrition and symptomatic HIV disease are associated with increased mortality [13,14,32].

There was no difference in the impact of 2 RDA multiple micronutrients or the standard of care on anthropometric measurements and CD4 cell count. This lack of difference could be due to the same factors discussed here.

There were no major adverse events observed from our study, and this was similar to what other micronutrient supplementation studies have reported [16,19,33].

The loss to follow up was higher than anticipated and this could have influenced the study outcome. The children lost to follow up were more likely to be underweight than those who completed the study. Similarly, there were a higher proportion of CRP-positive children among those lost to follow up than among those who completed the study. The implications of these two findings are not clear, but indicate that those lost to follow up were more ill than those who completed the study [34].

At one of the regional sites, we included children who were living in internally displaced camps in northern Uganda (Lira), and some of these relocated to distant areas while still in the study. However, the overall loss to follow up was comparable to what other micronutrient supplementation studies in the HIV population have reported [35].

## Conclusions

Twice the recommended dietary allowance of 14 micronutrients compared with 1 RDA of six multivitamins given as the "standard of care" for six months was well tolerated with no serious adverse events reported, but did not significantly alter mortality, growth or CD4 counts in HIV-infected children aged one to five years. Patients on HAART had a considerably lower mortality compared to those without. Future intervention studies should carefully consider: (1) the composition and dosing of the supplements; and (2) the power needed to detect a difference between arms.

## Competing interests

The authors declare that they have no competing interests.

## Authors' contributions

GN, TT and JKT participated in the conception, design and implementation of the study, statistical analysis, interpretation and drafting of the manuscript. CMN participated in design and implementation of the study. All authors read and approved the final manuscript.
